# Temperature Effect on the Nanostructure of SDS Micelles in Water

**DOI:** 10.6028/jres.118.008

**Published:** 2013-04-11

**Authors:** Boualem Hammouda

**Affiliations:** National Institute of Standards and Technology, Gaithersburg, MD 20899

**Keywords:** aggregation number, critical micelle temperature, ellipsoidal particles, material balance equations, micelle structure, SANS, SDS, small-angle neutron scattering, sodium dodecyl sulfate, surfactant

## Abstract

Sodium dodecyl sulfate (SDS) surfactants form micelles when dissolved in water. These are formed of a hydrocarbon core and hydrophilic ionic surface. The small-angle neutron scattering (SANS) technique was used with deuterated water (D_2_O) in order to characterize the micelle structure. Micelles were found to be slightly compressed (oblate ellipsoids) and their sizes shrink with increasing temperature. Fits of SANS data to the Mean Spherical Approximation (MSA) model yielded a calculated micelle volume fraction which was lower than the SDS surfactant (sample mixing) volume fraction; this suggests that part of the SDS molecules do not participate in micelle formation and remain homogeneously mixed in the solvent. A set of material balance equations allowed the estimation of the SDS fraction in the micelles. This fraction was found to be high (close to one) except for samples around 1 % SDS fraction. The micelle aggregation number was found to decrease with increasing temperature and/or decreasing SDS fraction.

## 1. Introduction

Surfactants are amphiphilic molecules that form micelles in aqueous medium. The hydrophilic charged head groups associate with water while the hydrophobic tails aggregate inside the micelle core. Many investigations used characterization methods such as scattering or microscopy in order to study micelle structures. Small-angle neutron scattering (SANS) studies from sodium dodecyl sulfate (SDS) micelles in water were conducted using mixtures of deuterated and non-deuterated surfactant molecules as well as end-deuterated molecules. Contrast variation series yielded micelle size, shape and aggregation number. Selective deuteration of the methyl groups gave insight into the packing of the micelle core [1]. Another SANS study of SDS micelle structure in water was conducted [2] in which the concentration and temperature dependences of the aggregation number were obtained. Lithium and sodium dodecyl sulfate (LDS and SDS) micelle formation in water was investigated upon LCl or NaCl salt addition. The aggregation number was found to increase with salt addition. NaCl was found to be more effective at screening Coulomb interactions than LiCl [3].

In another study, differential scanning calorimetry was used to determine the phase diagram for the SDS/water system [4]. The critical micelle formation (temperature and concentration) conditions were mapped out using calorimetry. The critical micelle concentration (CMC) of SDS in water was found to correspond to 0.2 % mass fraction which is equivalent to a molarity of 0.008 mol/L. For the studied SDS mass fraction range above 30 %, hydrated SDS crystals were observed below 25 °C. Cubic, hexagonal and lamellar phases along with a number of mesophases in-between were observed respectively for increasing SDS fractions. The hydrated SDS crystals observed at low temperatures melt to form micelles when temperature is increased. The Kraft point (corresponding to the intersection of the CMC with the crystal solubility limit) was found to be located around 10 °C. In yet another study, cryo-TEM images showed broad band-like aggregation of multiconnected threads forming at the air-solution interface as well as cylindrical micelles in the bulk of the solution. Small size micelles were found at high temperature and low SDS fraction while more extended structures were found at low temperature and high SDS fraction [5]. Sodium alkyl sulfates mixed surfactants form micelles just like each of the individual pure surfactants in solution. SANS measurements pointed to spherical and ellipsoidal micelle particles. The aggregation number for the mixed surfactant micelles lies closer to that for the longer chain surfactant. Moreover, the fractional charge of the mixed micelles was found to be smaller than that for each single-component [6].

SANS was also used to examine the structure of micelles formed of pure SDS or pure dodecyltrimethylammonium bromide (DTAB) in aqueous solution [7]. Micelles were found to be of oblate ellipsoidal shape. Using model fitting, minor and major axes sizes were determined. Upon addition of NaBr salt, a disk-to-tablet transition was observed. Salt effect seems to stretch the micelles into sheets. The short dimension scales with the size of the SDS hydrocarbon chain. Another investigation focused on mixtures of SDS and DTAB in aqueous solution with salt addition [8]. Interesting structures were uncovered. Unilamellar vesicles, then oligolamellar vesicles, then lamellar sheet structures were observed with increasing surfactant fraction. Another more recent study looked at SDS/DTAC (chloride) surfactant mixtures instead with added salts [9]. Similar results were found for DTAB or DTAC. Using bromide or chloride ions does not make much difference. Specific ionic salts were added to mimic the surfactant head groups in order to investigate the competition between simple ionic screening and ionic binding (attachment to the micelle surface). In another study, SDS was used to coat carbon nanotubes in order to enhance their solubility and prevent their aggregation [10].

Focus in the present paper is on the use of the SANS technique to investigate the structure of pure SDS micelles in aqueous medium and follow micelle changes with SDS fraction and sample temperature; a broader range in SDS fraction and sample temperatures were measured.

## 2. Samples and Characterization Method

Sodium dodecyl sulfate (SDS) surfactant (99 % purity) was purchased from Sigma-Aldrich (St. Louis, USA) and D_2_O (d-water) was purchased from Cambridge Isotope Labs (99.9 % purity).^1^ A series of SDS solutions were prepared for small-angle neutron scattering (SANS) measurements. Samples with the following SDS mass fractions were prepared: 0.1 %, 0.5 %, 1 %, 2 %, 5 %, 10 %, and 20 %. Three more samples were prepared where NaCl salt fraction was varied (0.1 mol/L, 0.2 mol/L and 0.5 mol/L) for the 1 % SDS/d-water sample. Samples were allowed to equilibrate overnight.

SANS measurements were made using the NG3 30 m SANS instrument at the NIST Center for Neutron Research. Temperature was varied between 10 °C and 90 °C with 10 °C intervals. In practice, the heating system lags behind slightly so that the actual measured sample temperatures are: 11 °C, 21 °C, 30 °C, 40 °C, 49 °C, 59 °C, 68 °C, 78 °C, and 87 °C. Standard overhead runs such as from the empty cell, the blocked beam as well as sample transmission and empty cell transmission runs were taken. SANS data were scaled to an absolute cross section using the empty beam transmission method. Standard data reduction method was used in order to obtain radially averaged intensity (units of cm^−1^) as function of scattering variable Q (units of Å^−1^).

## 3. Trends Observed in SANS Data

**Fig. 1:**
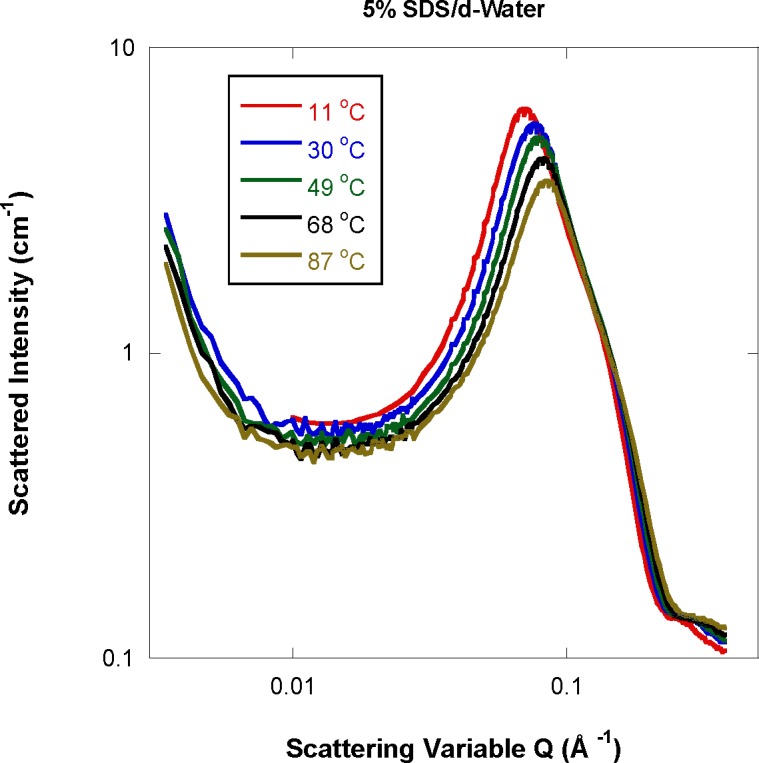
SANS data for 5 % SDS mass fraction while varying temperature. The peak and shoulder features are characteristic of ellipsoidal micelles.

SANS data show a weak low-Q (long-range) feature and a dominant intermediate-Q (shorter-range) feature which is due to the micelle particles structure. The intermediate-Q peak and shoulder features observed in the SANS data are characteristic of anisotropic micelles such as ellipsoidal particles in agreement with previous results [7]. These are seen to move to higher Q (Fig. 1) upon heating implying that particles get smaller with increasing temperature. The low-Q feature (observed at low SDS fractions) is likely due to clustering and characterizes water-soluble (especially ionic) systems. It has been discussed in the literature [11,12]. It is characteristic of mass fractals (Porod exponents between 2 and 3).

**Fig 2:**
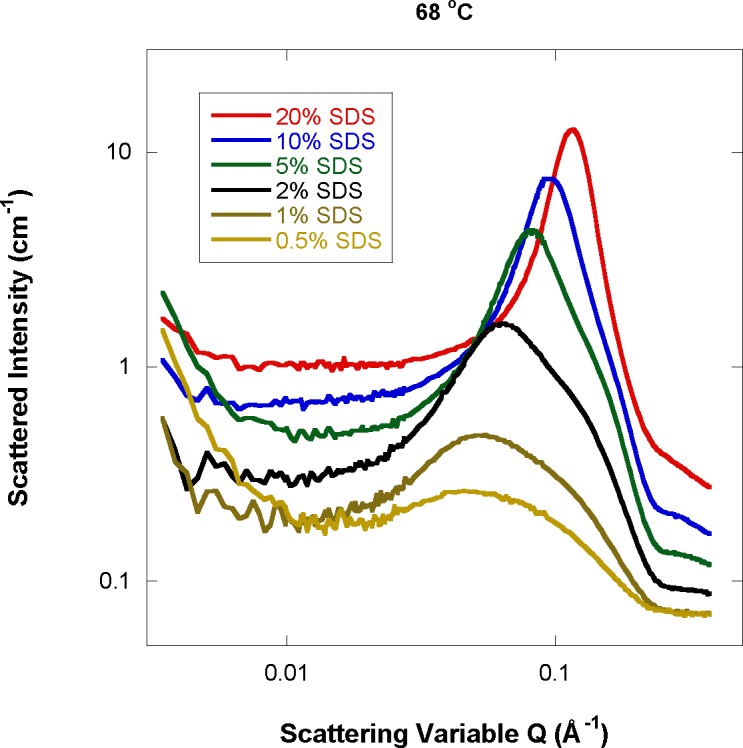
SANS data for varying SDS mass fraction and a fixed temperature of 68 °C.

Increasing the SDS mass fraction for fixed high temperature (68 °C) shows smooth shape change for the ellipsoidal micelles. Only samples that are above the critical micelle formation concentration (i.e., at or above 0.5 %) are included in this trend. The peak and shoulder features become more pronounced and move to higher Q (Fig. 2), which means that the micelles become more ellipsoidal and their packing gets tighter.

**Fig. 3:**
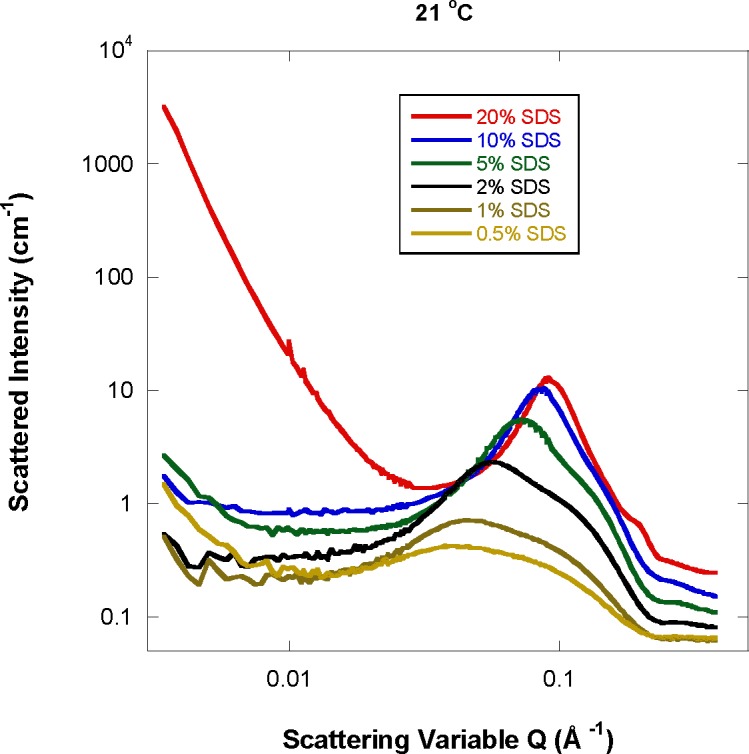
SANS data for varying SDS mass fraction and a fixed temperature of 21 °C.

At the fixed low temperature of 21 °C, increasing the SDS mass fraction affects the SANS data more drastically. The low-Q feature is not pronounced till the SDS mass fraction becomes high enough (Fig. 3). This feature characterizing long-range correlations between micelles becomes overwhelming for the 20 % SDS mass fraction sample. This 20 % SDS sample seems to contain another phase at 21 °C and below. The strong low-Q feature, smooth intermediate-Q peak variation and appearance of a high-Q shoulder are clues pointing to a two-phase system containing micelle particles as well as very large droplets. Visual inspection of this sample shows no macroscopic phase separation (no meniscus). Since no low-Q Guinier region was observed, the size of such droplets could not be estimated.

**Fig. 4:**
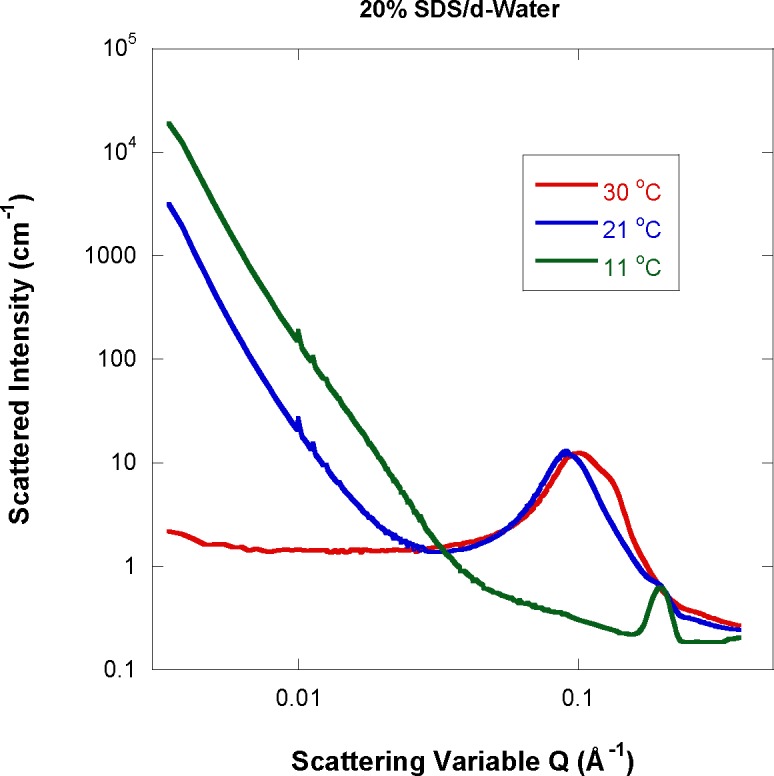
SANS data for the three lowest temperatures for the 20 % SDS sample.

In order to investigate the low-Q feature further, SANS data for the 20 % SDS sample are plotted for the first three temperatures in Fig. 4 (for 11 °C, 21 °C, and 30 °C). This feature is non-existent at 30 °C and is increasingly pronounced at lower temperatures. At 11 °C, a Bragg peak appears at high-Q. It is believed that at 11 °C, hydrated SDS crystals form in agreement with the previously reported phase diagram [4] while at higher temperatures, elliposoidal shape micelles dominate and SDS crystals have melted down. The Bragg peak defines the inter-lamellar d-spacing between the SDS-rich layers in the hydrated crystals separated by d-water layers.

In order to obtain quantitative information, SANS data are fitted to a realistic scattering model described next.

## 4. Scattering Model

The recurring clues characterizing the SANS data consist of two size scales observed on the intermediate-Q peak. This points to ellipsoidal shape micelles as reported previously for similar systems [6,9]. A scattering model consisting of a solution of interacting ellipsoidal particles is used to fit the SANS data. The scattering cross section is expressed as:

(1) $${\left[\frac{d\sum \left(Q\right)}{d\Omega }\right]}_{\text{ellipsoids}}=\Delta \rho^{2}\phi V_{P}P(Q)S_{I}(Q).$$

Here ∆ρ^2^ is the contrast factor, ϕ is the particle volume fraction, V_P_ is the particle volume, P(Q) is the single-particle form factor, and S_I_(Q) is the inter-particle structure factor. This model works best for spherical particles, and is used here for ellipsoidal particles that are not too distorted.

The form factor represents an average over orientations of the anisotropic particles. It involves the following integral:

(2) $$P(Q)=\frac{1}{2}\int_{-1}^{+1}d\mu P(Q,\mu ).$$

Here μ = cos(θ) has been defined where θ is the angle between the main axis of the ellipsoid and the $\vec{Q}$ direction. Particles are assumed to be ellipsoidal with half axes R_a_ and R_b_. For an oblate ellipsoid particle (with R_b_ > R_a_), an effective radius R_e_ is defined as:

(3) $$R_{e}{}^{2}=R_{b}{}^{2}+\mu^{2}(R_{a}{}^{2}-R_{b}{}^{2}).$$

The form factor amplitude is the same as the one for a sphere of radius R_e_:

(4) $$P(Q,\mu )={\left[\frac{3j_{1}({\mathrm{QR}}_{e})}{{\mathrm{QR}}_{e}}\right]}^{2}.$$

Here j_1_(QR_e_) is the spherical Bessel function of order 1. Note that the orientations of single particles are assumed to be decoupled (valid for not too distorted particles and not too high particle fraction). With this caveat, the Mean Spherical Approximation (MSA) is used to model the structure factor S_I_(Q). This model is known to be reliable when screened Coulomb interactions are present (such as for ionic micelles), and relies on the MSA closure relation to solve the Ornstein-Zernike Eq. (13). It should be mentioned that the approximate MSA model is often used since it relies on an analytical solution. Fits to this model yield effective sizes.

The following model parameters are used: ε is the dielectric constant, D is the micelle (also called macroion) effective diameter, κ is the Debye-Huckel inverse screening length, and z_m_e is the electric charge on the micelle surface where e is the electron charge.

The Debye-Huckel screening parameter (inverse length) squared is expressed as follows:

(5) $$\kappa^{2}=\frac{e^{2}}{\varepsilon k_{B}T}\left(z_{m}\frac{\phi }{V_{P}}+\frac{\phi_{\text{salt}}}{V_{\text{salt}}}\right)$$

ϕ and ϕ_salt_ are the micelle particle and salt volume fractions, V_P_ and V_salt_ are the particle and salt molecule volumes, and k_B_T is the sample temperature in absolute units.

The micelle volume fraction ϕ is expressed in terms of the number density $\bar{N}$ and micelle volume V_P_ = πD^3^/6 as $\phi ={\bar{N}V}_{P}$.

The MSA formalism used to derive the structure factor [13] is not reproduced here. This model is included in small-angle scattering data analysis software packages such as the IGOR-based package used at the NIST Center for Neutron Research [14].

Note that the MSA model was originally introduced for spherical particles and is used here for ellipsoidal particles. This approximate approach should be reliable when the intermicelle distance is large compared to the micelle size.

In order to perform fits to the SANS data when sample temperature was varied, tabulated temperature dependence of the dielectric constant for d-water [15] is used (i.e., is fixed to help the fits).

## 5. SANS Data Analysis

The model used to fit the SANS data consists of the sum of two functional forms: a low-Q power law function and the ellipsoidal micelles model:

(6) $$I(Q)=\frac{A}{Q^{n}}+{\left[\frac{d\sum \left(Q\right)}{d\Omega }\right]}_{\text{ellipsoids}}+B.$$

n is a low-Q Porod exponent, [dΣ(Q)/dΩ]_ellipsoids_ was discussed above and B is a constant representing the Q-independent background mostly due to incoherent scattering from hydrogen.

Smearing of the model was performed first using the SANS instrument resolution function. Then, nonlinear least-squares fits were performed on all SANS data sets. Fitting was reasonable in most cases despite the large number of fitting parameters. The resulting model parameters are: the low-Q scale factor A and Porod exponent n, the micelles volume fraction ϕ_fit_, the ellipsoidal micelles half axes R_a_ and R_b_, the scattering length density inside the micelles ρ_m_, the scattering length density for the solvent ρ_s_, and the charge on the micelles. The sample temperature in absolute units was also fixed as well as the dielectric constant for d-water [15]. The contrast factor involves the difference Δρ^2^ = (ρ_m_−ρ_s_)^2^ where ρ_m_ and ρ_s_ are the micelles and solvent scattering length densities respectively. Note that only this relative difference is relevant here.

**Fig. 5:**
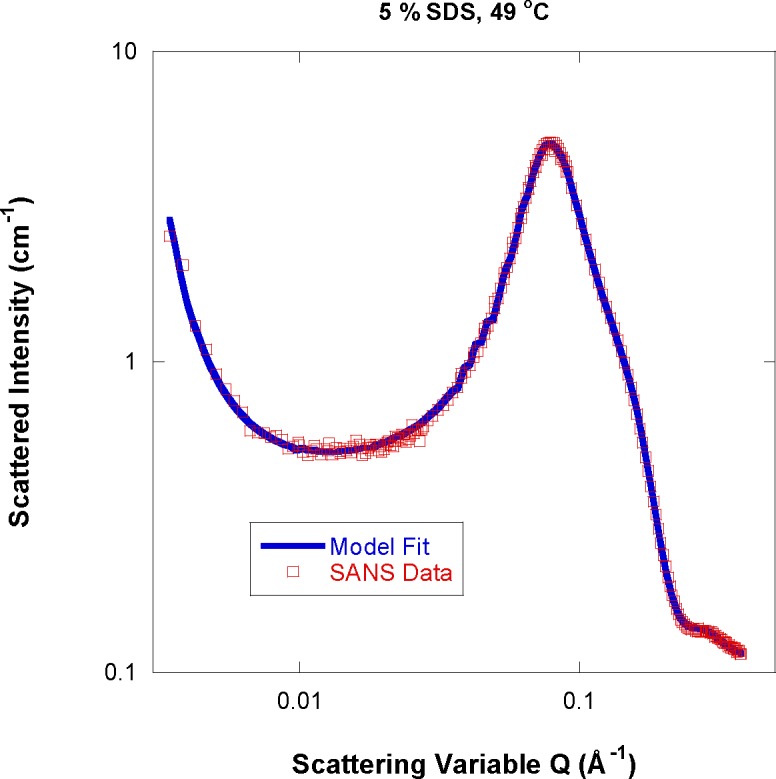
Typical model fit and SANS data for the 5 % SDS/d-water sample at 49 °C.

A typical fit is shown in Fig. 5 for the 5 % SDS mass fraction sample at 49 °C. The model used to fit reproduces the low-Q power law feature as well as hugs the intermediate-Q curve representing the oblate ellipsoidal micelles. The low-Q clustering feature is observed in most water-soluble systems [11,12].

**Fig. 6:**
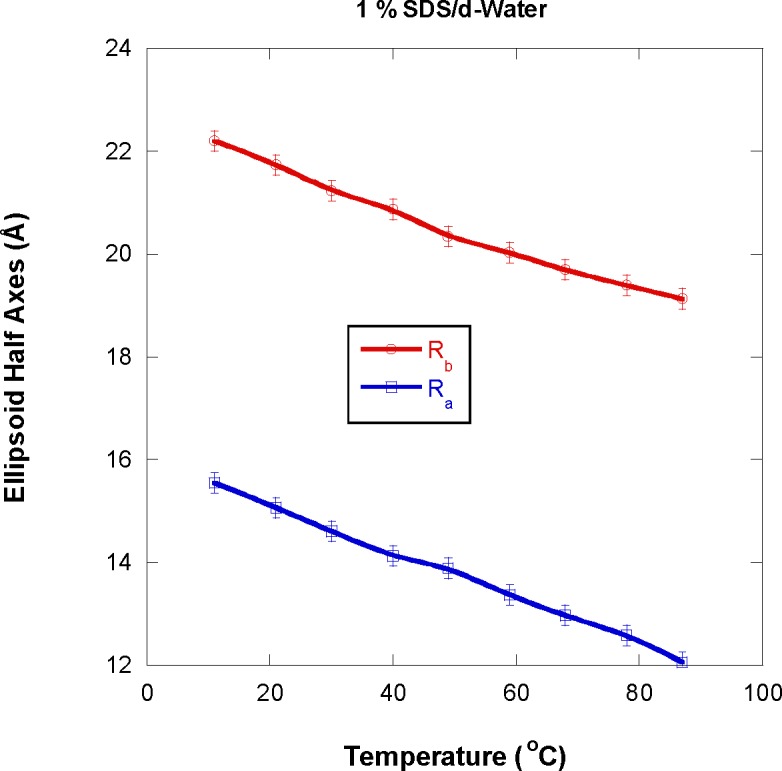
Variation of the ellipsoid micelles half axes with increasing temperature for the 1 % SDS sample. The lines going through the points are guides to the eye (smooth fitting).

Both ellipsoidal micelles half axes R_a_ and R_b_ decrease with increasing temperature as shown in Fig. 6. The value of R_b_ was systematically larger than R_a_ pointing to oblate (i.e., compressed) ellipsoidal micelles as expected [7,10]. Note that throughout this paper, error bars represent statistical precision and correspond to one standard deviation.

**Fig. 7:**
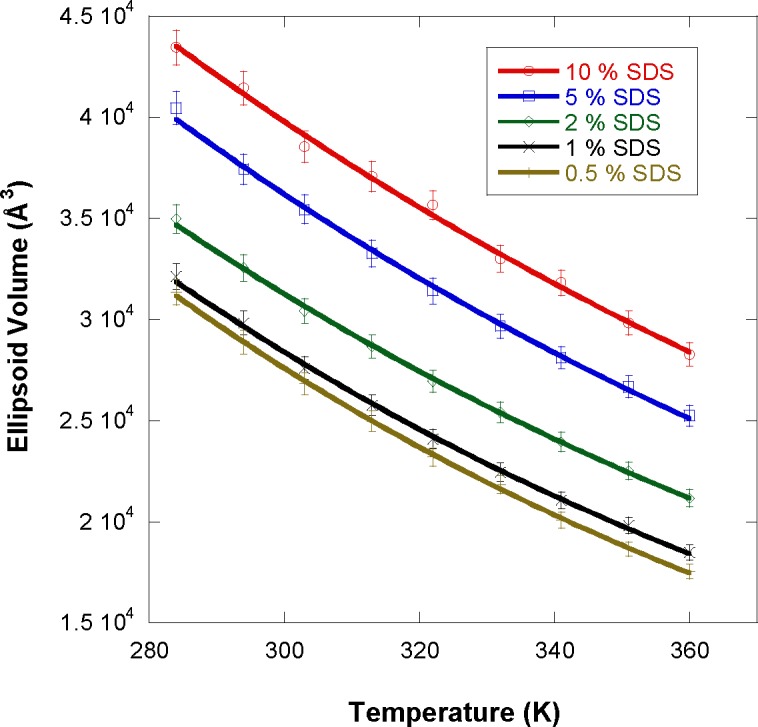
Variation of the ellipsoid micelle volume with increasing temperature for the various SDS mass fractions.

The ellipsoidal micelle (oblate scattering particle) volume is estimated as V_P_ = 4πR_a_R_b_^2^/3. This volume is seen (in Fig. 7) to decrease consistently with increasing temperature and to increase with increasing SDS mass fraction. As temperature increases, the micelle volume decreases (so does the aggregation number) thereby yielding more (smaller) micelles. This is likely due to many factors that include softening of hydrogen-bonding of water molecules to the surfactant headgroups and packing of the surfactant tails.

**Fig. 8:**
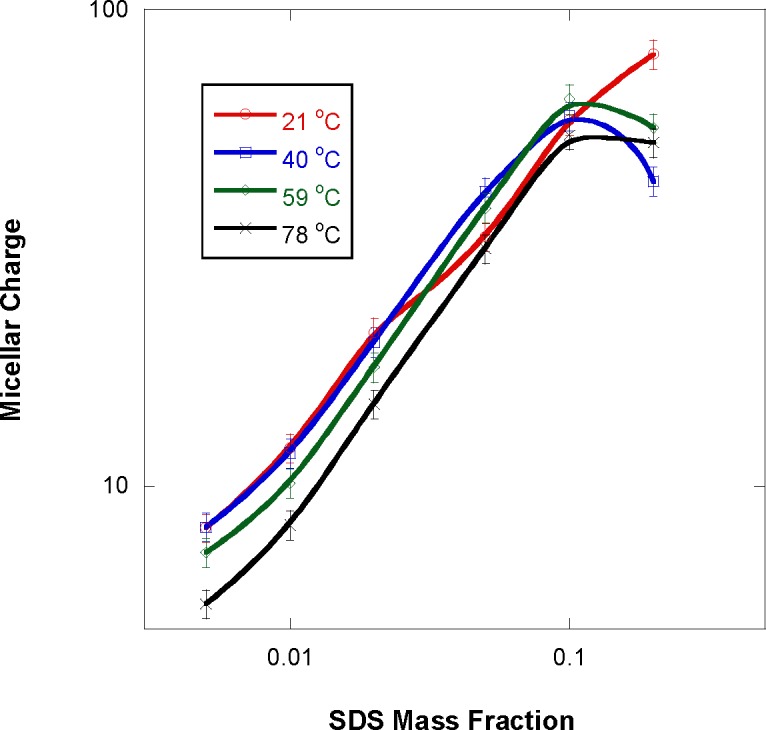
Variation of the micelle charge with increasing SDS mass fraction for various sample temperatures.

Fit results show that the charge on the micelles increases with SDS weight fraction (in Fig. 8) as it should since the size of micelles increases with increasing SDS fraction. Micelle charges, however, decrease with increasing temperature since the micelle volume decreases with increasing temperature. This trend breaks down for the highest SDS mass fraction (20 %) sample. The same saturation trend at high SDS fraction was observed previously [2]. Since the SDS molecule carries one electron charge on the ionized oxygen atom, the micelle charge scales with the micelle aggregation number.

**Fig. 9:**
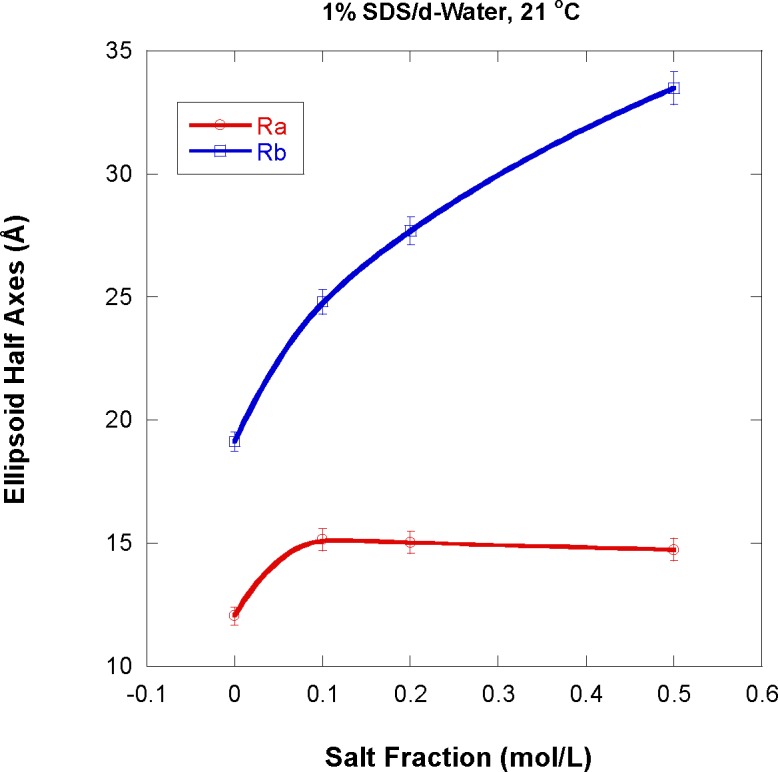
Variation of the ellipsoid micelles half axes with increase in NaCl salt content.

Sodium chloride (NaCl) salt was added to the 1 % SDS/d-water sample. Three salt fractions corresponding to 0.1 mol/L, 0.2 mol/L, and 0.5 mol/L were measured besides the no-salt (0 mol/L) sample. The minor half axis R_a_ is seen to increase, then flatten out (even slightly decrease) with increasing salt content while the major half axis R_b_ systematically increases (almost doubles from 0 mol/L to 0.5 mol/L salt content) as shown in Fig. 9. Salt tends to screen charges on the micelle surface and to neutralize charges in the solvent. The observation that neutralized micelles are larger is not surprising since these are characterized by weaker Coulomb interactions that tend to repel SDS molecules from each other. Upon salt addition, the oblate micelles seem to grow laterally with not much increase in their thickness.

Fit results are reliable except for the 0.1 % SDS fraction sample. For all other samples, the ellipsoidal micelles volume fraction (from the fits) ϕ_fit_ is systematically lower than the SDS sample mixing volume fraction ϕ_mix_ (which is equal to the SDS mass fraction divided by its density which is around 1.01 g/cm^3^). This means that not all SDS material takes part in micelle formation. Some of it remains homogeneously dissolved in the solvent. The temperature dependence is rather weak. Data for the lowest (0.1 %) and highest (20 %) SDS mass fraction samples break the trend. It should be mentioned that the measured contrast factor ∆ρ^2^ follows a similar trend.

This last result suggests that some SDS molecules remain homogeneously dissolved in the solvent and do not participate in micelle formation (except for very low SDS fractions) and prompts a closer look. Material balance equations are derived next in order to estimate the relative SDS fraction that participates in micelle formation.

## 6. Material Balance Equations

The measured samples were prepared by mixing a volume fraction ϕ_mix_ of SDS in D_2_O. The fits of SANS data to the model described earlier yielded micelle volume fractions ϕ_fit_ that are either close to or lower than ϕ_mix_ which implies that not all the SDS molecules are used to form the micelles. The SDS fraction found in the micelles is denoted f_SDS_ while the fraction remaining dissolved in D_2_O is 1−f_SDS_. Similarly, we assume that a (yet undetermined) fraction $f_{D_{2}O}$ of d-water is included in the micelles while the $1-f_{D_{2}O}$ fraction remains dissolved. Denoting n_SDS_ and $n_{D_{2}O}$ the total number of SDS and D_2_O molecules in the sample (of volume V), the number of SDS and D_2_O molecules in the micelles are therefore n_SDS_f_SDS_ and $n_{D_{2}O}f_{D_{2}O}$ respectively.

The SDS molecules are formed of H(CH_2_)_12_OSO_3_^−^ negatively charged ions that either form the micelles or remain dissolved in the solvent and Na^+^ counterions that are homogeneously distributed in the solvent. We use the notation v_SDS_, v_Na_, and $v_{D_{2}O}$ for the SDS (negative macroions only), sodium counterions and D_2_O molecular volumes, N_m_/V for the micelles number density, b_SDS_, b_Na_, and $b_{D_{2}O}$ for the scattering lengths of the SDS, sodium and D_2_O molecules respectively. Note that the scattering length density is defined as the ratio of scattering length over volume. The micelle volume is called v_m_.

A set of material balance equations is derived in order to account for the content of each sample and its scattering length density.

1. Micelle volume
  (7) $$v_{m}=\frac{f_{\mathrm{SDS}}n_{\mathrm{SDS}}v_{\mathrm{SDS}}}{N_{m}}+\frac{f_{D_{2}O}n_{D_{2}O}v_{D_{2}O}}{N_{m}}$$
2. Micelle volume fraction obtained from the fit
  (8) $$\phi_{\mathrm{fit}}=\frac{N_{m}v_{m}}{V}$$
3. Sample mixing volume fraction for SDS
  (9) $$\phi_{\mathrm{mix}}=\frac{n_{\mathrm{SDS}}{(v}_{\mathrm{SDS}}+v_{\mathrm{Na}})}{V}$$
4. Sample mixing volume fraction for D_2_O
  (10) $$1-\phi_{\mathrm{mix}}=\frac{n_{D_{2}O}v_{D_{2}O}}{V}$$
5. Scattering length density difference between the micelles and the solvent
  (11) $$\Delta \rho =\frac{n_{\mathrm{SDS}}f_{\mathrm{SDS}}b_{\mathrm{SDS}}+n_{D_{2}O}f_{D_{2}O}b_{D_{2}O}}{N_{m}v_{m}}-\frac{n_{\mathrm{SDS}}(1-f_{\mathrm{SDS}}{)b}_{\mathrm{SDS}}+n_{D_{2}O}(1-f_{D_{2}O}{)b}_{D_{2}O}+n_{\mathrm{SDS}}b_{\mathrm{Na}}}{V-N_{m}v_{m}}$$

Material balance equations 2) to 4) are used to replace N_m_, $\frac{n_{\mathrm{SDS}}v_{\mathrm{SDS}}}{V}$, and $\frac{n_{D_{2}O}v_{D_{2}O}}{V}$ in terms of ϕ_fit_ and ϕ_mix_ leaving the following pair of equations (keeping the same numbering):

(12) $$\begin{array}{l} 1)\;1=\frac{\phi_{\mathrm{mix}}}{\phi_{\mathrm{fit}}}\frac{1}{1+v_{\mathrm{Na}}/v_{\mathrm{SDS}}}f_{\mathrm{SDS}}+\frac{\left(1-\phi_{\mathrm{mix}}\right)}{\phi_{\mathrm{fit}}}f_{\mathrm{SDS}}\\ 5)\;\Delta \rho =C*f_{\mathrm{SDS}}+D*f_{D_{2}O}+E \end{array}$$

Material balance eq 1) yields $f_{\mathrm{SDS}}=A+B*f_{D_{2}O}$

The various linear coefficients have been defined as:

(13) $$\begin{array}{l} A=\frac{\phi_{\mathrm{fit}}}{\phi_{\mathrm{mix}}}(1+v_{\mathrm{Na}}/v_{\mathrm{SDS}})\\ B=-\frac{\left(1-\phi_{\mathrm{mix}}\right)}{\phi_{\mathrm{mix}}}(1+v_{\mathrm{Na}}/v_{\mathrm{SDS}})\\ C=\frac{\phi_{\mathrm{mix}}}{1+v_{\mathrm{Na}}/v_{\mathrm{SDS}}}\frac{b_{\mathrm{SDS}}}{v_{\mathrm{SDS}}}\frac{1}{\phi_{\mathrm{fit}}(1-\phi_{\mathrm{fit}})}\\ D=(1-\phi_{\mathrm{mix}})\frac{b_{D_{2}O}}{v_{D_{2}O}}\frac{1}{\phi_{\mathrm{fit}}(1-\phi_{\mathrm{fit}})}\\ E=-\frac{\phi_{\mathrm{mix}}}{1+v_{\mathrm{Na}}/v_{\mathrm{SDS}}}\frac{b_{\mathrm{SDS}}}{v_{\mathrm{SDS}}}\frac{1}{1-\phi_{\mathrm{fit}}}-(1-\phi_{\mathrm{fit}})\frac{b_{D_{2}O}}{v_{D_{2}O}}\frac{1}{1-\phi_{\mathrm{fit}}}+\frac{\phi_{\mathrm{mix}}}{1+v_{\mathrm{Na}}/v_{\mathrm{SDS}}}\frac{b_{\mathrm{Na}}}{v_{\mathrm{SDS}}}\frac{1}{1-\phi_{\mathrm{fit}}} \end{array}$$

These two linear equations with two unknowns (f_SDS_ and $f_{D_{2}O}$ are solved to yield:

(14) $$\begin{array}{l} f_{D_{2}O}=\frac{\Delta \rho -\mathrm{CA}-E}{\mathrm{CB}+D}\\ f_{\mathrm{SDS}}=A+{\text{Bf}}_{D_{2}O} \end{array}$$

The molecular volumes and scattering lengths for SDS, D_2_O and Na are used (Table 1) as inputs in order to calculate the SDS and D_2_O number fractions found in the micelles. Random mixing was assumed throughout.

This approach assumes that all Na^+^ ions are located in the solvent with no fraction bound to the micelle surface. It also assumes that the molecular volumes remain constant with temperature.

**Fig. 10:**
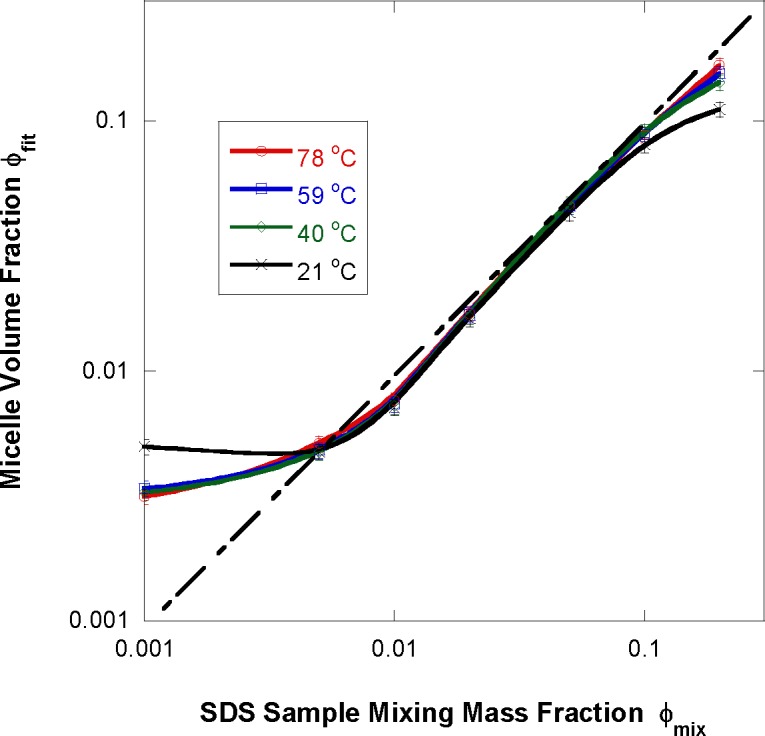
Variation of the fitted micelle volume fraction with the SDS sample mixing mass fraction at various temperatures. The dashed line represents a slope of one (equal fractions).

**Fig. 11:**
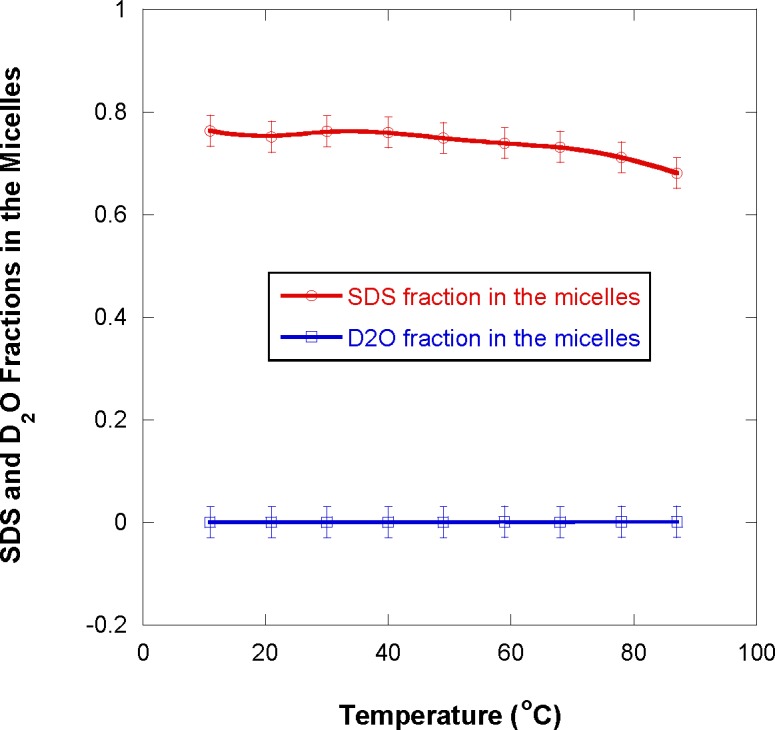
Fractions of the SDS and D_2_O molecules found in the micelles for the 1 % sample.

The SDS fraction in the micelles f_SDS_ was found to be close to 1 except for the 1 % SDS fraction sample for which it is around 80 % (Fig. 11). This result shown in Fig. 11 is consistent with Fig. 10 and with the following argument. Micelles form when the SDS fraction reaches the critical micelle concentration (CMC). Added surfactant goes to forming the micelles while the surfactant remaining dissolved (not participating in micelle formation) remains close to the CMC fraction of 0.2 % (8 mmol/L). For the 1 % SDS fraction sample, the fraction that remains dissolved corresponds to 20 % of the content (0.2 %/1 % = 20 %). The D_2_O fraction in the micelles $f_{D_{2}O}$ was found to be 0 for all samples (within statistical precision). It is comforting to see that most SDS is used to form the micelles and that no D_2_O can be found inside the micelle core which contains hydrophobic tails. This result is in agreement with a previous investigation [1] in which water was found not to penetrate the hydrocarbon core (beyond the first CH_2_ group). This result for the SDS ionic micelles investigated here is, however, at variance with a previously reported investigation of nonionic Pluronic micelles for which some D_2_O was found inside the micelle core [16]. Pluronics are formed of poly(propylene oxide) and poly(ethylene oxide) blocks. The PPO blocks become hydrophobic at high temperature thereby forming micelles while the PEO blocks remain dissolved in the micelllar corona. The corona contains a large amount of water while the micelle core was found to contain a small amount of water (probably around the oxygen sites on the PPO blocks). The SDS micelles discussed here have a completely hydrophobic tail which forms the micelle core which is free of water.

Note that the SDS fraction in the micelles f_SDS_ decreases with temperature probably due to the softening of the micelle formation driving interactions (due to hydrophobic tails and hydrophilic ionic heads). Note also that these results for f_SDS_ and $f_{D_{2}O}$ are independent of the micelle volume v_m_. The aggregation number (number of SDS molecules per micelle), on the other hand, does depend on v_m_. It is expressed as:

(15) $$N_{\text{agg}}=\frac{f_{\mathrm{SDS}}n_{\mathrm{SDS}}}{N_{m}}=f_{\mathrm{SDS}}\frac{\phi_{\mathrm{mix}}}{\phi_{\mathrm{fit}}}\frac{v_{m}}{v_{\mathrm{SDS}}{(1+v}_{\mathrm{Na}}/v_{\mathrm{SDS}})}.$$

**Fig. 12:**
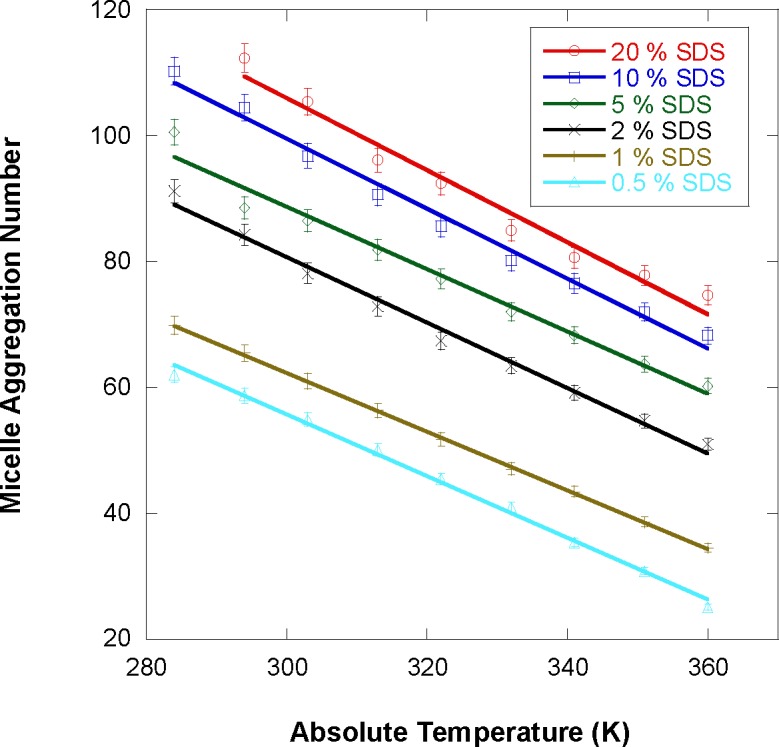
Variation of the micelle aggregation number with temperature for the various SDS sample fractions. Linear fits are included.

The aggregation number (N_agg_) is plotted in Fig. 12 for varying temperature for the various SDS sample mixing fractions. N_agg_ is seen to decrease with increasing temperature since the micelle sizes (and volume) decrease with increasing temperature. The micelles number density N_m_/V = ϕ_fit_/v_m_, however, increases with increasing temperature. Moreover, N_agg_ increases with increasing SDS fraction as it should.

## 7. Summary and Discussion

This research focused on an old topic and reported new results. The SDS surfactant forms micelle structures in aqueous medium. Micelle particles were found to be mostly of an oblate ellipsoidal shape (compressed spheroid). Nonlinear least squares fits to an appropriate model corresponding to non-dilute mixtures of oblate spheroids yielded estimates for the minor and major micelle half axes. The 1 % SDS sample at 40 °C, for example, is characterized by half axes of 14.1±0.1 Å and 20.9±0.1 Å respectively. These sizes are comparable with the previously reported dimensions of 12.0 Å and 20.3 Å for the same system [7]. At the highest SDS fraction of 20 % and lowest measured temperature of 11 °C, another phase was observed. The two characteristic clues of a Bragg peak at high-Q and a strong low-Q signal point to hydrated SDS crystals in agreement with the published phase diagram [4].

The estimated micelle ellipsoid volume was found to decrease with increasing temperature and/or decreasing SDS fraction. Moreover, the micelle charge was also found to decrease with increasing temperature and/or decreasing SDS fraction as it should.

The oblate ellipsoid micelles half axes were found to increase with increasing NaCl salt addition. The minor half axis increases slightly then flattens out at 15 Å beyond 0.1 mol/L salt while the major half axis keeps on increasing up to 33 Å for 0.5 mol/L salt. The minor size is comparable to the SDS hydrocarbon tail size (fully extended size around 17 Å). Salt addition seems to screen charges on the micelles surface thereby allowing micelles to grow laterally while remaining of the thickness of one SDS close-to-stretched molecule.

The fitted micelle volume fraction ϕ_fit_ scales with the sample mixing SDS volume fraction ϕ_mix_ except at low (lower than 0.5 %) and high (higher than 10 %) fractions. In the intermediate region around 1 % SDS fraction, it was concluded that not all SDS molecules participate in micelle formation; a small fraction remains homogeneously dissolved. In order to estimate that fraction and to assess whether any water gets into the micelle core, material balance equations were derived. The discrepancy between ϕ_fit_ and ϕ_mix_ as well as the fitted scattering length density difference between the micelle core and the solvent ∆ρ are used as inputs in order to back out the SDS fraction participating in micelle formation. For the 1 % SDS sample, at least 80 % of the SDS molecules are found to participate in micelle formation. The remaining 20 % are used to keep the dissolved SDS close to the CMC level. Moreover, no water was found inside the micelle core region. The micelles aggregation number (number of SDS molecules per micelle) was found to decrease with increasing temperature and with decreasing SDS fraction. This scales well with the observed variation of the micelle volume and surface charge.

The reported results are in agreement with other findings in the literature. New results include detailed characterization of the micelle structure and its variation with temperature, mass fraction and salt content. A set of material balance equations was introduced and found to be important for the understanding of subtle changes in the micelle content. Such material balance equation may prove to be important in characterizing hydration in globular proteins.

## Figures and Tables

**Table 1:** Tabulated volumes and scattering lengths used as input in order to solve the material balance equations. The scattering length unit is the Fermi (10^−13^ cm).

	Chemical Formula	Mass (g/mol)	^3^ Volume (cm)	Density (g/cm^3^)	Scattering Length (Fermi)
SDS Ion	H(CH_2_)_12_OSO_3_^−^	265	v_SDS_=4.36*10^−22^	d_SDS_=1.01	b_SDS_=12.34
Sodium	Na^+^	23	v_Na_=3.94*10^−23^	d_Na_=0.971	b_Na_=3.63
d-water	D_2_O	20	$v_{D_{2}O}=3*10^{-23}$	$d_{D_{2}O}=1.11$	$b_{D_{2}O}=19.145$

## Footnotes

1 Certain commercial equipment, instruments, or materials are identified in this paper to foster understanding. Such identification does not imply recommendation or endorsement by the National Institute of Standards and Technology, nor does it imply that the materials or equipment identified are necessarily the best available for the purpose.
